# Reporter Cell Lines for the Characterization of the Interactions between Human Nuclear Receptors and Endocrine Disruptors

**DOI:** 10.3389/fendo.2015.00062

**Published:** 2015-05-11

**Authors:** Marina Grimaldi, Abdelhay Boulahtouf, Vanessa Delfosse, Erwan Thouennon, William Bourguet, Patrick Balaguer

**Affiliations:** ^1^IRCM, Institut de Recherche en Cancérologie de Montpellier, Montpellier, France; ^2^U1194, INSERM, Montpellier, France; ^3^Université Montpellier, Montpellier, France; ^4^ICM, Institut Régional du Cancer de Montpellier, Montpellier, France; ^5^U1054, INSERM, Montpellier, France; ^6^CNRS UMR5048, Centre de Biochimie Structurale, Montpellier, France

**Keywords:** nuclear receptors, environmental-disrupting compounds, reporter cell lines

## Abstract

Endocrine-disrupting chemicals (EDCs) are exogenous substances interfering with hormone biosynthesis, metabolism, or action, and consequently causing disturbances in the endocrine system. Various pathways are activated by EDCs, including interactions with nuclear receptors (NRs), which are primary targets of numerous environmental contaminants. The main NRs targeted by environmental contaminants are the estrogen (ER α, β) and the androgen (AR) receptors. ERs and AR have pleiotropic regulatory roles in a diverse range of tissues, notably in the mammary gland, the uterus, and the prostate. Thus, dysfunctional ERs and AR signaling due to inappropriate exposure to environmental pollutants may lead to hormonal cancers and infertility. The pregnane X receptor (PXR) is also recognized by many environmental molecules. PXR has a protective role of the body through its ability to regulate proteins involved in the metabolism, the conjugation, and the transport of many exogenous and endogenous compounds. However, the permanent activation of this receptor by xenobiotics may lead to premature drug metabolism, the formation, and accumulation of toxic metabolites and defects in hormones homeostasis. The activity of other NRs can also be affected by environmental molecules. Compounds capable of inhibiting or activating the estrogen related (ERRγ), the thyroid hormone (TRα, β), the retinoid X receptors (RXRα, β, γ), and peroxisome proliferator-activated (PPAR α, γ) receptors have been identified and are highly suspected to promote developmental, reproductive, neurological, or metabolic diseases in humans and wildlife. In this review, we provide an overview of reporter cell lines established to characterize the human NR activities of a large panel of EDCs including natural as well as industrial compounds such as pesticides, plasticizers, surfactants, flame retardants, and cosmetics.

## Introduction

Human nuclear receptors (NRs) are a family of 48 transcription factors, many of which have been shown to be activated by endogenous ligands. NRs regulate cognate gene networks involved in key physiological functions such as cell growth and differentiation, development, homeostasis, or metabolism (1, 2). As a consequence, inappropriate exposure to environmental pollutants, which have the ability to substitute for natural ligands, can cause proliferative, reproductive, and metabolic disorders, including hormone-dependent cancers, infertility, diabetes, or obesity.

**Figure 1:**
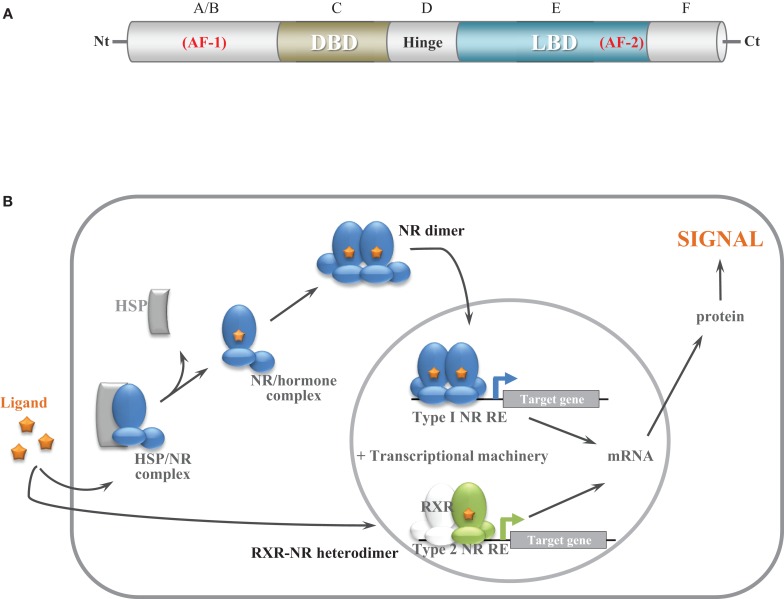
**General nuclear receptor structure and function**. **(A)** Structural organization of NRs. NRs comprise six domains, including a N-terminal activation function domain, a central DNA binding domain, and a C-terminal ligand-binding domain carrying a ligand-dependant transcriptional function. **(B)** Schematic model of NR function. Before ligand binding, type I NRs form inactive complexes with chaperone proteins in the cytoplasm (AR) or in the nucleus (ERs) whereas type II NRs (RXR heterodimers) are bound to their target genes with corepressors. Ligand binding results in the dissociation of chaperone proteins and binding and activation to target genes for type I NRs. Ligand binding results in corepressors release and coactivator recruitment for type II NRs.

NRs are transcriptional regulators comprising several domains, including a N-terminal activation function domain (AF-1), a central DNA-binding domain (DBD), and a C-terminal ligand-binding domain (LBD) carrying a ligand-dependent transcriptional activation function (AF-2) (2) (Figure 1A). When unassociated with their ligand, type I NRs form inactive complexes with chaperone proteins in the cytoplasm, whereas type II NRs are located in the nucleus and bind to the DNA response elements of their target genes along with corepressors (Table 1; Figure 1B). Ligand binding triggers major conformational changes in the receptor LBD that lead to the dissociation of chaperones and corepressors, nuclear translocation and DNA binding of type I NRs, and recruitment of coactivators, thus initiating gene transcription. In presence of agonists in the ligand-binding pocket, corepressors dissociate and the recruitment of transcriptional coactivators is favored (3–5). Reciprocally, interaction with antagonists avoids association with coactivators and enables corepressors recruitment (3–5). The LBD also contributes to the modulation of the N-terminal AF-1 through interdomain crosstalk, which enable AF-1 and AF-2 domains to recruit coactivators individually or in a synergistic manner (6–8).

**Table 1:** **Nuclear receptor characterization**.

NR	Cellular localization in absence of ligand	Active form
ERα (NR3A1)	Nuclear	Homodimer
ERβ (NR3A2)	Nuclear	Homodimer
AR (NR3C4)	Cytoplasmic	Homodimer
ERRγ (NR3B3)	Nuclear	Monomer
PPARα (NR1C1)	Nuclear	RXR heterodimer
PPARγ (NR1C3)	Nuclear	RXR heterodimer
TRα (NR1A1)	Nuclear	RXR heterodimer
TRβ (NR1A2)	Nuclear	RXR heterodimer
PXR (NR1I2)	Nuclear	RXR heterodimer
RXRα (NR2B1)	Nuclear	Heterodimer or homodimer
RXRβ (NR2B2)	Nuclear	Heterodimer or homodimer
RXRγ (NR2B3)	Nuclear	Heterodimer or homodimer

Endocrine-disrupting chemicals (EDCs) are exogenous substances that interfere with the function of hormonal systems and produce a range of developmental, reproductive, neurological, immune, or metabolic diseases in humans and wildlife (9, 10). Many EDCs are man-made chemicals produced by industry and released into the environment as, for example, bisphenols, phthalates, pesticides, organotins, flame retardants, polychlorinated biphenyls, dioxins, or alkylphenols. EDCs can also be naturally produced by plants or fungus, like the genistein and daidzein phytoestrogens, or the zealenone mycoestrogen. Population exposure to EDCs is very variable in both quantity and quality according to the area they live in. Agricultural and industrialized areas are typically prone to contamination by a broad range of chemicals that may seep into the soil and groundwater. Living organisms are being exposed to these chemicals through ingestion of contaminated food and water, breathing of contaminated air, or direct contact with a contaminated soil. People working with pesticides, fungicides, and industrial chemicals are particularly exposed to these toxic substances and thus have a higher risk of developing reproductive or endocrine disorders. EDCs affect the endocrine system of organisms in various ways, like, for instance, by mimicking natural hormones activity, antagonizing their action, or modifying their synthesis, metabolism, and transport. Pathways activated by these substances include stimulation of membrane receptors and the aryl hydrocarbon receptor, and stimulation of enzymatic machineries implicated in hormone biosynthesis/metabolism. However, the majority of reported harmful effects of EDCs have been attributed to their interference with hormone signaling mediated by nuclear receptors (11–14). Most original studies have focused on NRs involved in reproductive processes, in particular ERs and AR, but recent data have shown that EDCs can act as nano- to micromolar ligands for many other receptors including the activity of pregnane X receptor (PXR), ERRγ, TRs, retinoid X receptors (RXRs), PPARα, or PPARγ.

The need to screen thousands of chemicals for their human NRs interactions leads several laboratories including ours to develop robust reporter gene assays with high sensitivity, selectivity, and responsiveness for NR ligands. Unlike other *in vitro* techniques including ligand-binding assays and endogenous gene expression measurement by quantitative RT-PCR, the stable expression of reporter genes creates robust and reproducible and easy-to-handle cellular models, which are easy transferable from one laboratory to another. Here, we review recent studies in which we have characterized NR activity of EDCs using reporter cells.

## Estrogen Receptors, Estrogen-Responsive Reporter Cell Lines, and Environmental Estrogens

ERα (NR3A1) and ERβ (NR3A2) are nuclear receptors for the sex hormone 17β-estradiol (E2), which play an important role in the growth and maintenance of various tissues such as the uterus, mammary gland, bones, or the cardiovascular system. Those ERs are broadly distributed throughout the body and display both distinct and overlapping expression patterns in tissues (15). Indeed, ERα is preferentially expressed in the uterus, kidney, liver, and heart, whereas ERβ is primarily expressed in the ovary, prostate, gastrointestinal tract, lung, bladder, and hematopoietic and central nervous systems (16). However, ERα and ERβ are also coexpressed in numerous tissues such as the mammary gland, adrenal, thyroid, bones, and some regions of the brain.

Interestingly, when ERs are coexpressed, ERβ exhibits an inhibitory action on ERα-mediated gene expression (17, 18) so that ERβ has been shown to antagonize several ERα-mediated effects including fat reduction and cellular proliferation in breast, uterus, or prostate (19–21). It is thus pertinent to ask whether EDCs have different selectivities for ERs. Although the LBDs of ERα and ERβ share a high degree of homology in their primary amino acid sequence and are very similar in their tertiary architecture (4), some ERα- and ERβ-selective pharmaceutical ligands have been identified (22).

**Figure 2:**
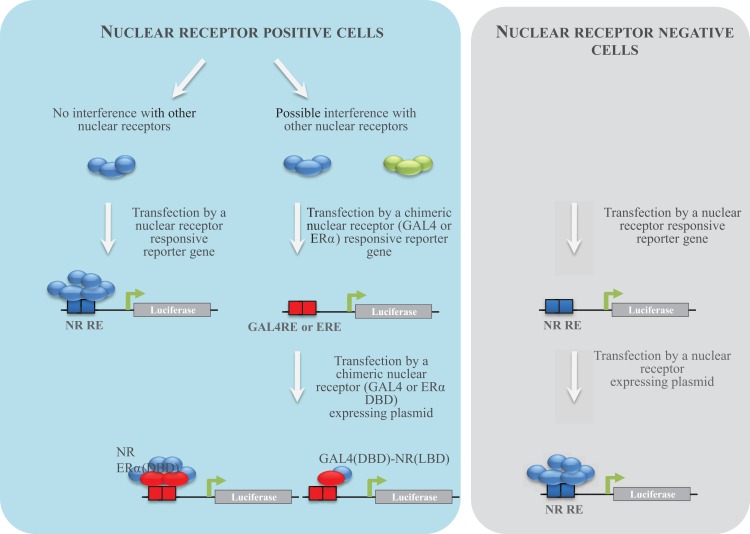
**NR reporter cell establishment strategy**. Different strategies to establish reporter cell lines have been used. The first consisted to transfect NR-positive cells with a NR-responsive reporter gene. When several NRs are able to activate the same promoter in a cell, an alternative strategy consists to transfect a chimeric NR-expressing plasmid. Cells are transfected by plasmids enabling the expression of the chimeric construct of the yeast GAL4 DBD fused to the NR LBD and the luciferase under the control of GAL4. Cells (ER negative) can also be transfected by a plasmid enabling the expression of a chimeric NR in which the DBD was replaced by the one of ERα and the luciferase gene under the control of estrogens. The third strategy consisted to transfect NR-negative cells in a first step by an NR-responsive reporter gene and in a second step by an NR-expressing plasmid.

To characterize human ER activity of chemicals, different reporter cell lines have been generated by the scientific community (Table 2; Figure 2). The first strategy consisted of stably transfecting breast (MCF-7, T47-D) or ovarian cancer (BG1) cells, which express endogenously ERα with an estrogen-regulated luciferase gene (23–26). These cell lines were extensively used to measure ERα activity of pure chemicals or environmental samples. However, because they do not express ERβ, another strategy consisted of expressing ERα or ERβ in ER-negative cell lines (23, 27–29). In our case and in order to obtain comparable cell lines, we first transfected the estrogen-responsive reporter gene in Hela cells, which does not express ERs. In a second step, cells stably transfected with the ERE-luciferase plasmid (HELN cells) were transfected with an ERα or ERβ construct (23). Using these cell lines, we have characterized the ERα and ERβ potency of ER environmental ligands. These molecules are highly heterogenous and include few high affinity ligands (EC_50_ values between 10 pM and 1 nM) (Table 3). These potent estrogens are pharmaceutical agents contained in contraceptive pills (ethinyl estradiol, hexestrol), human estrogens (estradiol, estrone, estriol) (30), or the mycoestrogen zearalenone and its metabolites (31). Many other environmental compounds interact with ERs with medium to low affinity (EC_50_ values between 1 nM and 10 μM) (Table 3). Phytoestrogens are plant-derived substances that have estrogenic activity (16). Genistein, the principal phytoestrogen in soy, is an agonist for both ERs, with, however, a marked preference for ERβ (27, 30). Some pesticides like dichloro-diphenyltrichloroethane (DTT), methoxychlore, chlordecone, vinclozolin, and their metabolites act as estrogenic chemicals. Interestingly, chlordecone and methoxychlor display ERα agonistic but ERβ antagonistic activity. Finally, cosmetics like conservative parabens and UV-screens benzophenones, and many industrial compounds such as bisphenols and their halogenated derivatives, alkylphenols, and phthalates display estrogenic activity (32). For these compounds, the affinity for ERs is closely dependent of their structure. The estrogenic potency of parabens is clearly dependent of the alkyl chain length. Propyl and butyl parabens are more active than methyl and ethyl parabens (33). Similarly, alkylphenols with long chain (C8–C9) have better affinity for ERs than alkylphenols with short chain. In a similar manner, the number and the position of the hydroxyl groups of benzophenones have a strong impact on their potency (34). Finally, the nature of additional groups of bisphenols is also very important for the estrogenic activity of these compounds (35–37). As an example, Bisphenol S of which the two phenolic groups are linked by a sulfur dioxide (SO_2_) group is 100-fold less potent for ERs than Bisphenol AF of which the phenolic groups are linked by a C(CF3)_2_ group (35).

**Table 2:** **Nuclear receptor reporter cell lines developed to screen EDCs**.

NR	Cell type	Active NR	Reporter gene	Reference
ERα (NR3A1)	MCF-7	hERα	ERE-β-globin-luciferase	(23)
	T47-D	hERα	ERE_3_-TATA-luciferase	(26)
	BG1	hERα	ERE_3_-TATA-luciferase	(25)
	BG1	hERα	ERE_3_-TATA-luciferase	(24)
	U2OS	hERα	ERE_3_-TATA-luciferase	(29)
	293	hERα	ERE-MMTV-phosphatase	(27)
	HS578T	hERα	ERE_3_-TATA-luciferase	(28)
	HeLa	hERα	ERE-β-globin-luciferase	(23)
	HeLa	ΔAB-hERα	ERE-β-globin-luciferase	(30)

ERβ (NR3A2)	U2OS	hERβ	ERE_3_-TATA-luciferase	(49)
	293	hERβ	ERE-MMTV-phosphatase	(27)
	HS578T	hERβ	ERE_3_-TATA-luciferase	(28)
	HeLa	hERβ	ERE-β-globin-luciferase	(23)
	HeLa	ΔAB-hERβ	ERE-β-globin-luciferase	(30)

AR (NR3C4)	PC3	hAR, hGR	MMTV-Luciferase	(41)
	MDA-MB-453	hAR, hGR	MMTV-Luciferase	(40)
	U2OS	hAR	ARE_3_-TATA-luciferase	(49)
	HeLa	hARα ERα (DBD)	ERE-β-globin-luciferase	(35)

ERRγ (NR3B3)	HeLa	GAL4 (DBD)-hERRγ (LBD)	GALRE_5_-β-globin-luciferase	(35)

PPARα (NRC1)	HeLa	GAL4 (DBD)-hPPARα (LBD)	GALRE_5_-β-globin-luciferase	(78)

PPARβ (NRC2)	HeLa	GAL4 (DBD)-hPPARβ (LBD)	GALRE_5_-β-globin-luciferase	(78)

PPARγ (NRC3)	U2OS	hPPARγ1	PPARRE_3_-TATA-luciferase	(77)
	U2OS	hPPARγ2	PPARRE_3_-TATA-luciferase	(77)
	HeLa	GAL4 (DBD)-hPPARγ (LBD)	GALRE_5_-β-globin-luciferase	(78)

TRα (NR1A1)	GH3	*rTRα, *rTRβ	DR4_2_-TATA-luciferase	(82)
	PC12	**cTRα	DR4_4_-TATA-luciferase	(83)
	HeLa	GAL4 (DBD)-*rTRα (LBD)	GALRE_5_-β-globin-luciferase	(84)

TRα (NR1A2)	HeLa	GAL4 (DBD)-*rTRβ (LBD)	GALRE_5_-β-globin-luciferase	(84)

PXR (NR1I2)	HepG2	hPXR	CYP3A4-luciferase	(96)
	HepG2	hPXR	CYP3A4-luciferase	(97)
	HepG2	hPXR	CYP3A4-luciferase	(98)
	HepG2	hPXR	CYP3A4-luciferase	(99)
	HeLa	GAL4 (DBD)-hPXR (LBD)	GALRE_5_-β-globin-luciferase	(100)

RXRα (NR2B1)	HeLa	GAL4 (DBD)-mRXRα (LBD)	GALRE_5_-β-globin-luciferase	(108)

WT, Wild type NR; ΔAB, AB domain-deleted NR; ERα DBD, NR within the DBD were replaced by the hERα DBD; GAL4 DBD-NR LBD, chimeric NR constituted by the yeast GAL4 DBD fused to the NR LBD; MMTV, mouse mammary tumor virus; CYP3A4, cytochrome P4510 3A4; *r, rat; **chicken.

**Table 3:** **EDCs and their NR targets**.

EDCs	NR targets	EC50 range	Lead compound	Nature
Mycoestrogens	ERα (NR3A1)	0.01–1 nM	Zearalenone	Full agonist
	ERβ (NR3A2)	0.01–1 nM	Zearalenone	Partial agonist
	AR (NR3C4)	1–10 μM	Zearalenone	Antagonist
	PXR (NR1I2)	1–10 μM	Zearalenone	Full agonist

Phytoestrogens	ERα (NR3A1)	0.1–1 μM	Genistein	Full agonist
	ERβ (NR3A2)	0.01–0.1 μM	Genistein	Partial agonist

Parabens	ERα (NR3A1)	1–10 μM	Butyl paraben	Full agonist
	ERβ (NR3A2)	1–10 μM	Butyl paraben	Full agonist

Benzophenones	ERα (NR3A1)	0.1–1 μM	Benzophenone-2	Full agonist
	ERβ (NR3A2)	0.1–1 μM	Benzophenone-2	Full agonist
	AR (NR3C4)	1–10 μM	THB	Antagonist

Bisphenols	ERα (NR3A1)	0.01–1 μM	BPA	Partial agonist
	ERβ (NR3A2)	0.01–1 μM	BPA	Partial agonist
	AR (NR3C4)	0.01–1 μM	BPA	Antagonist
	ERRγ (NR3B3)	0.001–0.1 μM	BPA	Agonist
	PXR (NR1I2)	1–10 μM	BPA	Agonist

Halogenated bisphenols	ERα (NR3A1)	0.1–10 μM	TetrachloroBPA	Partial agonist
	ERβ (NR3A2)	0.1–10 μM	TetrachloroBPA	Partial agonist
	PPARγ (NR1C3)	1–10 μM	TetrabromoBPA	Partial agonist
	TRα (NR1A1)	1–10 μM	TetrabromoBPA	Antagonist
	TRβ (NR1A2)	1–10 μM	TetrabromoBPA	Antagonist

Alkylphenols	ERα (NR3A1)	0.01–1 μM	4-tert-Octylphenol	Agonist
	ERβ (NR3A2)	0.01–1 μM	4-tert-Octylphenol	Partial agonist
	AR (NR3C4)	1–10 μM	4-tert-Octylphenol	Antagonist
	ERRγ (NR3B3)	1–10 μM	4-tert-Octylphenol	Antagonist
	PXR (NR1I2)	1–10 μM	4-tert-Octylphenol	Agonist

Phthalates	ERα (NR3A1)	1–10 μM	BBP	Agonist
	ERβ (NR3A2)	1–10 μM	BBP	Partial agonist
	PPARα (NR1C1)	1–100 μM	MEHP	Agonist
	PPARγ (NR1C3)	1–100 μM	MEHP	Agonist

Perfluorinated compounds	PPARα (NR1C1)	1–100 μM	PFOA	Agonist
	PPARγ (NR1C3)	1–100 μM	PFOA	Agonist

Pesticides	ERα (NR3A1)	0.1–10 μM	2,4′-DDE	Agonist
	ERβ (NR3A2)	0.1–10 μM	2,4′-DDE	Partial agonist
	AR (NR3C4)	0.1–1 μM	M2 vinclozolin	Partial agonist
	PXR (NR1I2)	0.1–10 μM	Pretilachlor	Agonist

Organotins	PPARγ (NR1C3)	1–10 nM	TBT	Partial agonist
	RXRα (NR2B1)	1–10 nM	TBT	Agonist

*THB, trihydroxy-benzophenone; BPA, bisphenol A; BBP, benzyl butyl phthalate; MEHP, mono ethyl hexyl phthalate; PFOA, perfluorinated octanoic acid; DDE, dichlorodiphenyldichloroethylene; TBT, tributyltin*.

Interestingly, using HELN cell lines expressing N-terminal domain-deleted ERα and ERβ, we have shown that the agonistic efficacy of environmental estrogens depends on the receptor subtype and vary drastically among molecules from full agonists to weak agonists or antagonists. Whereas benzophenone-2 and 4-tert-alkylpenol acted as full agonists for both subtypes, ferutinine, α-zearalanol, bisphenol C (BPC), tetrachloro BPA (TCBPA), chlordecone, 2,2-bis(*p*-hydroxyphenyl)-1,1,1-trichloroethane (HPTE) and 2,4′ diphenyldichloroethylene (2,4′-DDE) are selective activators of ERα. On the contrary, BPA and butylparaben activated ERβ more efficiently than ERα. These results indicated that environmental estrogens also acted in a subtype-specific manner as full agonists, partial agonists, or antagonists by using different combinations of the N- and C-terminal activation functions of hERα and hERβ.

## Androgen Receptor, Androgen Responsive Reporter Cell Lines, and Environmental Anti-androgens

Androgen receptor (AR) (NR3C4) plays a crucial role in the regulation of target genes expression in physiological processes like development and differentiation of the male embryo and spermatogenesis initiation and maintenance, as well as neuro-endocrine system functioning (38). In the absence of ligands, AR is essentially localized in the cytoplasm. Binding to androgens enables HSPs dissociation and AR translocation to the nucleus. The AR LBD strongly contributes to the modulation of the N-terminal AF-1 through ligand-induced interdomain association. Furthermore, in AR, it appears that AF-1 predominates over AF-2 (39). The presence of androgens is essential for the regulation of male embryo development and differentiation processes and spermatogenesis initiation and maintenance. Like to estrogens, androgens influence also the development and growth of the mammary gland in women. Treatment of animals and cultured cells with androgens activates AR and has either inhibitory or stimulatory effects on genes transcription that are under steroid hormone control. Anti-androgens can disrupt this process.

To characterize human AR activity of chemicals, different reporter cell lines have been generated (Table 2). MDA-MB-453 (AR and GR positive) breast cancer cells have been transfected by the steroid responsive MMTV-luciferase plasmid (40). Two other reporter cell lines have been established by co-transfecting PC3 (41) and CHO (42) AR-negative cells with an MMTV-luciferase gene and an AR expressing plasmid. MDA-MB-453 (MDA-KB2) and PC3 (PALM) cells were extensively used to test anti-androgenicity of chemicals (31, 34, 36, 43–46) and environmental samples (47, 48). A problem with testing androgenicity is the activation of the reporter gene by GR, which is endogenously expressed in these cells. In order to improve the selectivity of the reporter cell line for testing androgenic agonist activity, van der Burg et al. (49) transfect U2OS cells, which express low amounts of GR with an androgen-responsive luciferase gene and an AR expressing plasmid (49). Another strategy developed by our group was to express in HELN (HeLa ERE-Luc) cells, a chimeric AR in which the DBD was replaced by the one of ERα (35). In these cells, only the AR ERα DBD is able to activate luciferase expression.

Using these reporter cell lines, we have measured the (anti)androgenicity of environmental compounds. Most of the compounds described to be estrogenic are also anti-androgenic. The most potent of them are zearalenone and some of its metabolites (36), M2 vinclozolin metabolite (44), 2,4′-DDE (50), 2,3,4-trihydroxy-benzophenone (THB) (34), and BPC (35) (Table 3).

## Estrogen-Related Receptor γ, ERRγ Reporter Cell Lines, and Their Environmental Ligands

The ERR subfamily of orphan receptors is closely related to ERs and includes three members, ERRα (NR3B1), ERRβ (NR3B2), and ERRγ (NR3B3) (51). ERRα is expressed at higher levels than the two other ERR subtypes and is detected in the heart, kidney, intestinal tract, skeletal muscle, and brown adipose tissue. ERRβ and ERRγ are mainly expressed in the heart and the kidney (52). It has been suggested that ERRs may play a central role in regulating energy metabolism (53). Meanwhile, the rise in the incidence of metabolic syndromes correlates with the increased use and distribution of industrial chemicals suspected of playing a role in generation of obesity (54). Altogether, these data suggest that EDCs and ERRγ may be involved in this epidemic crisis.

Additionally, it has been demonstrated that ERRγ can interfere with estrogen signaling (51, 55) by recognizing ERs DNA-binding elements and activating ERs target gene (56, 57). Expression of ERRγ is associated with favorable prognosis of breast cancer (58) and exogenous overexpression of ERRγ in a prostate cancer cell line inhibits proliferation (59). Furthermore, treatment with an ERRβ/γ agonist has been shown to promote this antiproliferative effect. To date, ERRγ has not been shown to interact with any physiologically relevant small molecules, suggesting that this receptor manifest constitutive activity (60, 61). Indeed, crystallographic analyses of ERRγ indicated that these receptors adopt the transcriptionally active conformation in the absence of any ligand (60).

To our knowledge, only our group established an ERRγ reporter cell line (Table 2). In order to characterize the interaction of environmental compounds with human ERRγ, we first developed a HeLa cell line expressing the luciferase gene under the control of the yeast GAL4 transcription factor (HG5LN cells). In a second step, these cells were stably transfected with a plasmid enabling the expression of the chimeric construct of the yeast GAL4 DBD fused to the ERRγ LBD (35). With this cell line, we were able to confirm that BPA, bisphenol E (BPE), and others phenols as medium (EC_50_ values in the 10–100 nM range) binders of ERRγ (62). To date, the other compounds screened for affinity or activity on ERRγ were known endocrine disruptors with estrogen-like activity. Most of them are not potent agonists (63) or antagonists (61, 64, 65). Their EC_50_ or IC_50_ values are in the micromoles range.

## Peroxysome Proliferator-Activated Receptors, PPAR-Responsive Reporter Cell Lines, and Environmental PPAR Ligands

The NR subfamily of peroxisome proliferator-activated receptors (PPARs) includes three members, PPARα (NR1C1), PPARβ/δ (NR1C2), and PPARγ (NR1C3). These receptors bind to PPAR-responsive DNA regulatory elements in the form of heterodimers with RXR. PPARs have distinct tissue distributions and physiological roles (66–68). PPARα is preferentially expressed in the heart, liver, and brown adipose tissue, whereas PPARβ/δ is ubiquitously expressed. They both play an important role as activators of fatty acid oxidation pathways and thus in the regulation of energy homeostasis. Furthermore, it has been shown that PPARα stimulates cholesterol catabolism, heme synthesis, and participates in the control of urea synthesis and amino acid metabolism. PPARβ/δ is involved in the control of cell proliferation and differentiation and is required for gut and placental development. PPARγ, for its part, is highly expressed in adipose tissues and plays a key role in regulating adipogenesis (69), lipid metabolism, and glucose homeostasis by improving insulin sensitivity (70). PPARs bind and respond to dietary fatty acids and various lipid metabolites, including eicosanoids, prostaglandins, and oxidized phospholipids (67, 71).

In accordance with their tissue distributions and roles as sensors of lipids/fatty acids levels, in regulating fatty acid catabolism, and in lipid storage, all three PPARs are thought to be strongly involved in the metabolic syndrome. However, in light of the particular role of PPARγ in adipose tissue development and maintenance, it has been suggested that the disruption of regulatory pathways controlled by PPARγ may be specifically implicated in the onset of diabetes and obesity (11). As a matter of fact, activation of PPARγ by some xenobiotic compounds has been shown to stimulate adipogenesis *in vitro* and *in vivo* by promoting the differentiation of preadipocytes of the fibroblastic lineage into mature adipocytes (72–75). This contributed to the “obesogen hypothesis” stating that the growing obesity epidemic due to the imbalance between caloric intake and expenditure could also implicate chemicals, so-called “obesogens,” which directly or indirectly increase fat accumulation and obesity (74, 76).

To characterize the human PPARγ activity of chemicals, Gijsbers et al. (77) stably transfected U2OS cells, which express low amounts of PPAR with a PPAR-responsive luciferase gene and PPARγ1- and PPARγ2-expressing plasmids (Table 2). In our group, we have developed a strategy similar to the one we developed for ERRγ. To obtain comparable cell lines, we transfected HG5LN cells (HeLa GAL4RE-luciferase) with plasmids expressing the LBD of the three human PPARs fused to the yeast GAL4 DBD (78). Using these cell lines, we were able to characterize the PPARγ activity of TCBPA and tetrabrominated BPA (TBBPA), perfluorooctanoic acid (PFOA), and mono(2-ethylhexyl)phthalate (MEHP) (37, 73). TBBPA and TCBPA activate partially PPARγ with approximately 100-fold less potency (EC_50_ values in the micromoles range) (Table 3) than the reference pharmaceutical compound rosiglitazone (EC_50_ value of 10 nM). Interestingly, while PFOA and MEHP are PPARα and PPARγ agonists (EC_50_ values in the 1–100 μM range), TBBPA, TCBPA, and their biotransformation products do not notably impact PPARα and PPARδ. Using these cells, we also characterized the RXR–PPARγ activity of organotins (72). To assess the specific effect of tributyltin (TBT) on RXR and PPARγ, cells were co-incubated with saturating concentrations of CD3254 (RXR agonist) or rosiglitazone (PPARγ agonist) and increasing concentrations of rosiglitazone, CD3254, or TBT. Like CD3254, TBT is able to further activate the rosiglitazone-saturated heterodimer. However, in contrast with rosiglitazone, TBT appears unable to act in conjunction with CD3254 to enhance the activity of RXR/PPARγ. TBT activates RXRα as efficiently as the full agonist CD3254, whereas it behaves as a very weak PPARγ agonist.

## Thyroid Receptors, Thyroid Responsive Reporter Cell Lines, and Environmental TR Ligands

The NR subfamily of thyroid receptors (TRs) includes two members, TRα (NR1A1) and TRβ (NR1A2). Their tissue distributions are relatively ubiquitous and the expression of these proteins begins early in development (79). Thyroid hormones (THs) are essential for the normal development, growth, and metabolism of all vertebrates (79) and play a major role in neurogenesis and brain function at all stages of development (80). THs are produced by the thyroid. Tetra-iodothyronine (thyroxine or T4) and tri-iodothyronine (T3) are the principal representatives of circulating THs. In target cells, T4 is converted to T3, which is the most active TH. Moreover, THs are key developmental and differentiation hormones in all organs of the body, including the central nervous system and the skeleton.

Several environmental chemicals can disturb the thyroid hormone system by affecting synthesis, transport, metabolism, and cellular uptake (81). To characterize chemicals acting at the TR level, different reporter cell lines have been generated (Table 2). Freitas et al. (82) stably expressed a thyroid-regulated luciferase gene in rat pituitary TR-positive cells (GH3). Jugan et al. (83) stably expressed a thyroid-regulated luciferase gene in rat PC12 cells previously transfected by an avian TRα-expressing plasmid. In our group, we developed a strategy similar to the one we used for ERRγ and PPARs reporter cells. HG5LN were transfected with plasmids expressing GAL4 (DBD)-human TRs (LBD) (84). Using these cell lines, we showed that BPA and its halogenated derivatives are TR antagonists in the 1–100 μM range (84) (Table 3). Among the other compounds that act through NR binding, Freitas et al. (82) showed that hydroxylated BDEs and PCBs are TR agonists in the 1–10 μM range.

## Pregnane X Receptor, PXR Responsive Cell Lines, and PXR Environmental Ligands

Pregnane X receptor (NR1I2) is a broad-specificity sensor playing a critical role in the regulation of phase I (CYP), phase II (conjugating), and phase III (ABC family transporters) detoxifying enzymes, coordinately regulating steroid, drug, and xenobiotic clearance in the liver and intestine (85). Activated PXR binds to gene promoters as a heterodimer with RXR and triggers target genes expression such as cytochrome P450 3A4 (CYP3A), UDP-glycosyltransferase (UGT1A1), and multidrug resistance protein 1 (MDR1) (86). PXR plays an important role in protecting the endocrine system from EDCs by sensing concentration increases of these chemicals and stimulating detoxification pathways, resulting in a decreased interaction of EDCs with other NRs. This PXR-driven elimination of xenobiotics confers a positive role to the activation of this NR. On the contrary, PXR activation can also prevent effects of hormones or drugs by stimulating prematurely their metabolism, which could lead to adverse interactions or harmful effects. Additionally, inactive compounds can be metabolized into active metabolites that could have deleterious consequences (87). On the other hand, the activation of PXR has been linked to an increased risk of cardiovascular (88), metabolic (89), and cancer diseases (90, 91). Unlike most NRs that tend to be specialized in binding few ligands with structural homologies, PXR binds a multitude of drugs such as the antibiotic rifampicin (92), the anti-cancer taxol (93), the anti-cholesterol SR12813 (94), the St John’s worth anti-depressor hyperforin (95), and many more, reviewed in di Masi et al. (86).

Since PXR is also able to bind environmental compounds, several groups have established reporter cell lines to study their interactions. Lemaire et al. (96), Ratajewski et al. (97), Raucy et al. (98), and Sekimoto et al. (99) have developed similar cellular models (Table 2). They are human hepatoma HEPG2 cells co-transfected with a human PXR expression vector and the luciferase gene driven by the human CYP3A4 promoter. Because we suspected that expression of PXR could reduce the potency of compounds that are metabolized by target genes of PXR (CYP3A4, UGT1A1, or MDR1), we expressed GAL4 (DBD)-PXR (LBD) in HG5LN cells (100). Using the HG5LN GAL4-PXR reporter cell line, we have shown that a large number of environmental chemicals like pesticides (101), natural and synthetic estrogens, alkylphenols (102, 103), and polychlorinated biphenyls (103) are targets of PXR. EC_50_ values of these compounds for PXR are generally in the 1–100 μM range excepted for some pesticides (pretilachlor, oxadiazon) exhibiting EC_50_ values are in the submicromolar range (101). We noticed that HG5LN GAL4-PXR cells are more sensitive for some chemicals (i.e., clotrimazol, transnonachlor) than the HEPG2 PXR CYP3A4 cells (96, 101) (Table 3). This is probably due to the fact that the PXR chimeric receptor is unable to activate the expression of detoxifying enzymes. On the contrary, in HEPG2 PXR cells, the ligand can activate PXR, which in turn increases its metabolism and reduce its intracellular concentration during the assay.

## Retinoid X Receptors, RXR Responsive Cell Lines, and RXR Environmental Ligands

The NR subfamily of RXRs includes three members, RXRα (NR2B1), RXRβ (NR2B2), and RXRβ (NR2B3). RXRs are particular since they represent heterodimerization partners for about one-third of NRs and are therefore implicated in the regulation of numerous signaling pathways in both ligand-dependent and ligand-independent manners (104). RXRs form three different types of dimers: RXR homodimer, permissive heterodimers, and non-permissive heterodimers. The so-called “permissive” RXR heterodimers are able to be activated when ligand binds to RXR, even in the absence of the partner receptor ligand. On the contrary, non-permissive heterodimers cannot be activated by the RXR ligand alone and RXR remain silent in absence of ligand for the partner NR. However, in both cases, it has been reported that RXR ligands and ligands of the partner receptors could act in a synergistic manner to activate heterodimers (1, 105). The involvement of RXR heterodimers in the regulation of multiple nuclear signaling pathways signifies that RXR ligands can potentially exert numerous harmful effects on human health. RXRs are activated by 9-*cis* retinoic acid as well as docosahexaenoic acid (106, 107).

Retinoid X receptor reporter cell lines established by co-transfection with the GAL4RE-luciferase and the LBD of the three mouse RXRs, fused to the yeast GAL4 DBD plasmids were established by Nahoum et al. (108) (Table 1). In our group, in order to determine if human RXR could also be activated by environmental chemicals, we used the RXR-permissive PPARγ reporter cell line. We thus demonstrated that TBT, triphenyltin, tripropyltin, and dibutyltin are able to activate RXR at nanomolar concentrations. Excepted organotins, we failed to identify environmental chemicals with RXR activity.

## Conclusion

Endocrine-disrupting chemicals are chemicals of great concern because these compounds, which are ubiquitously present in our daily environment, can cause adverse effects in humans and wildlife. By deregulation of NR-mediated transcription, EDCs can alter endocrine functions and cause infertility, malformations, metabolic troubles, or increase incidence of cancers. Though ERs are primary targets of EDCs, other members of the NR family, including AR, ERRγ, PPARs, TRs, RXRs, and PXR have been shown to correspond to secondary targets of EDCs. The weak structural relationships between EDCs and natural ligands make their interactions with NRs poorly understood and hardly predictable. Therefore, it is necessary to characterize the deleterious interactions between environmental compounds and NRs and develop robust screening methods.

*In vitro* and cell-based screens designed to identify NR ligands include binding assays using recombinant NR full length or LBD or transcriptional assays using cells with stable transfection of NR and a corresponding responsive luciferase gene. Cell-based assays have the advantage of typically being high-throughput, requiring less time and costs. In the present article, we review the reporter cell lines that have been established to characterize EDCs interaction with ERs, AR, ERRγ, PPARs, TRs, PXR, and RXRs. Characterization of the harmful interaction between these different NRs and environmental compounds is currently studied in several laboratories for the assessment of toxic potential of large numbers of chemicals.

Controversy remains about the EDCs mechanism of action and low-dose effect. Recent studies have revealed additional EDCs targets through which EDCs can stimulate rapid cellular responses at very low concentrations. These include membrane-associated NRs (109, 110) and the G protein-coupled receptor 30 (111). Development of robust *in vitro* screening methods for these new EDCs targets is also very important.

Most of our current knowledge of EDCs action is based on single molecule exposure in model systems *in vitro* or *in vivo*. These efforts have therefore taken little account of a more realistic situation in which humans are chronically exposed to low doses of multiple EDCs, which are likely to act in an additive, antagonistic, or synergistic manner through their combined actions on various nuclear and membrane-associated receptors. Indeed, a growing number of studies indicate that human risk assessment approaches based on single molecule exposure underestimate the risk for adverse effects of chemicals (112). Thus, one of the greatest future challenges in risk assessment is to develop novel protocols to evaluate the toxicity of complex mixtures of chemicals. In this regard, the robust *in silico* screening methods, which are currently being developed for the prediction of the harmful interaction between large numbers of chemicals and their cellular targets, will be of great value (35, 113–115).

## Conflict of Interest Statement

The authors declare that the research was conducted in the absence of any commercial or financial relationships that could be construed as a potential conflict of interest.
